# Supplementary material for: Risk of COVID-19 re-infection and its predictors (CORES): protocol for a community-based longitudinal cohort study in Vellore, India
Source: BMJ Open. 2022 May 24;12(5):e059869. doi: 10.1136/bmjopen-2021-059869 (PMC9130666; doi:10.1136/bmjopen-2021-059869)
Supplement: Author's manuscript [file bmjopen-2021-059869.draft_revisions.pdf]

BMJ Open is committed to open peer review. As part of this commitment we make the peer review history of every article we publish publicly available.

When an article is published we post the peer reviewers' comments and the authors' responses online. We also post the versions of the paper that were used during peer review. These are the versions that the peer review comments apply to.

The versions of the paper that follow are the versions that were submitted during the peer review process. They are not the versions of record or the final published versions. They should not be cited or distributed as the published version of this manuscript.

BMJ Open is an open access journal and the full, final, typeset and author-corrected version of record of the manuscript is available on our site with no access controls, subscription charges or pay-per-view fees (http://bmjopen.bmj.com).

If you have any questions on BMJ Open's open peer review process please email info.bmjopen@bmj.com

BMJ Open

Risk of COVID-19 re-infection and its predictors (CORES) – Study Protocol for a community based longitudinal cohort study

Journal:	*BMJ Open*
Manuscript ID	bmjopen-2021-059869
Article Type:	Protocol
Date Submitted by the Author:	04-Dec-2021
Complete List of Authors:	Madhavan, Ramya; Christian Medical College Vellore, The Wellcome Trust Research Laboratory<br>Paul, Jackwin; Christian Medical College Department of Community Health and Development<br>Babji, Sudhir; Christian Medical College and Hospital Vellore<br>Kumar, Dilesh; Christian Medical College Vellore<br>Prabhu, Savit; Christian Medical College and Hospital Vellore, Department of GI Sciences<br>Pulleri, Harsha; inStem<br>Annadorai, Ravikiran; inStem<br>Gowda, Sampreeth; inStem<br>John, Jacob; Christian Medical College and Hospital Vellore, Department of Community Health and Development<br>Kang, Gagandeep; Christian Medical College Vellore, Department of GI Sciences
Keywords:	COVID-19, IMMUNOLOGY, PUBLIC HEALTH

SCHOLARONE™
Manuscripts

Study Title:

Risk of COVID-19 re-infection and its predictors (CORES) – Study Protocol for a community based longitudinal cohort study

Authors' list and affiliations:

Joint First Authors

Ramya Madhavan*

Research Fellow

The Wellcome Trust Research laboratory

Division of GI Sciences

Christian Medical College, Vellore

India

E-mail: ramya.m@cmcvellore.ac.in

Jackwin Sam Paul*

Assistant Professor

Department of Community Health

Christian Medical College, Vellore

India

E-mail: jackwin.sam@cmcvellore.ac.in

*- Both authors contributed equally and would be joint first authors

Co-authors:

Sudhir Babji

Professor

The Wellcome Trust Research laboratory

Division of GI Sciences

Christian Medical College, Vellore

For peer review only

India

E-mail: sudhirbabji@cmcvellore.ac.in

Dilesh Kumar. A

Research Officer

The Wellcome Trust Research laboratory

Division of GI Sciences

Christian Medical College, Vellore

India.

E-mail: dilesh.kumara@cmcvellore.ac.in

Savit B Prabhu

Assistant Professor

The Wellcome Trust Research laboratory

Division of GI Sciences

Christian Medical College, Vellore

India.

E-mail: savit.b@cmcvellore.ac.in

Pulleri Kandi Harsha

Project Scientist II

Institute of Stem Cell science and Regenerative Medicine

Bangalore,

India

E-mail: harshapk@instem.res.in

Ravikiran Annadorai

Project Associate

Institute of Stem Cell science and Regenerative Medicine

For peer review only

Bangalore,
India
E-mail: ravikirana@instem.res.in
Gowda Sampreeth Ravi
Project Associate
Institute of Stem Cell science and Regenerative Medicine
Bangalore,
India
E-mail: sampreethg@instem.res.in
Jacob John
Professor
Department of Community Health
Christian Medical College, Vellore
E-mail: jacob@cmcsph.org
Corresponding author:
Gagandeep Kang
Professor of Microbiology
The Wellcome Trust Research laboratory
Division of GI Sciences
Christian Medical College, Vellore
India
E-mail: gkang@cmcvellore.ac.in

For peer review only

Abstract

Introduction

The incidence of SARS-CoV-2 re-infection has not been widely evaluated in Low and Middle-Income Countries (LMICs). Understanding immune responses elicited by SARS-CoV-2 natural infection and factors that lead to re-infection in a community setting is important for public health policy. We aim to investigate the risk of primary infection and re-infection among those without and with evidence of prior infection as defined by the presence of antibodies to SARS-CoV-2 spike protein.

Methods and Analysis

A baseline seroprevalence survey will test for SARS-CoV2 antibodies among 2000 randomly selected healthy adults in Vellore, India. Based on an expected seropositivity rate of 50% in the general population, with an annual attack rate of 12%, 6%, 4.8% and 4% among those unvaccinated and seronegative, vaccinated and seronegative, unvaccinated seropositives, and vaccinated seropositives respectively, we will recruit 1200 adults for follow up for a total of 24 months. Weekly self-collected saliva samples will be tested by RT-PCR to detect SARS-CoV2 infections, for a period of one year. For any person testing RT-PCR positive, blood samples will be collected within 2 days of RT-PCR positivity and on days 30 and 90 to assess IgG antibodies to the spike protein and for detailed immunogenicity to assess the kinetics and longevity of the antibody responses, B cell memory as well as T cell function and persistence post-infection. The data will be analyzed to estimate seroprevalence at baseline and over time, the risk factors for infection, rates of primary infection and re-infection and provide a comparison of the rates across groups based on infection and vaccination status.

Ethics and dissemination

The study has been approved by the institutional review board, (IRB No: 13585), Christian Medical College, Vellore.

For peer review only

For peer review only - http://bmjopen.bmj.com/site/about/guidelines.xhtml

Trial Registration

The trial has been registered with the Central Trial Registry of India (CTRI). The trial registration number is CTRI/2020/11/029438.

Strengths and limitations:

- Following up the cohort for a period of two years will help to capture the re-infection rates of SARS CoV 2 in the community.
- The use of saliva samples for SARS CoV 2 surveillance will be an acceptable alternate as it is self-directed, non-invasive.
- The cross reactivity between the SARS CoV 2 and other beta coronavirus will help in better understanding of the clinical outcome.
- The detailed immunogenicity following SARS CoV 2 infection will help in decision making with regards to booster vaccination.
- Though there is a good concordance of saliva and nasopharyngeal swab for SARS CoV 2 surveillance, there could be some cases which may be missed with saliva sampling.

<u>Study protocol</u>

Keywords

COVID-19, Immunology, Public Health.

<u>Background</u>

Immune responses to SARS-CoV-2 infection, vaccination, and the immune correlates of protection are areas of active investigation [1]. A few studies have shown that the development, amount, kinetics of antibodies may correlate with the clinical outcome of SARS CoV 2 infections. [2-5]. The coordinated response between humoral and cellular immunity has been hypothesized to be protective [6]. From a public health perspective, it is crucial to understand the duration of protective immunity offered by natural infections and vaccination [1]. There are

For peer review only

For peer review only - http://bmjopen.bmj.com/site/about/guidelines.xhtml

re-infections in patients who have recovered from the disease [7, 8]. At the population level, the incidence of re-infection over the long term (duration of one to two years) has not been evaluated, and this may not be feasible, given the rapid pace of vaccination. Preliminary studies suggest that antibody levels persist for at least seven to nine months or more post-infection [9-10]. The rates of attrition of potential immune correlates like memory B and T cell responses, and the association of these humoral and cellular immune parameters with subsequent re-infections are unknown. The duration of protective immunity to SARS-CoV-2 is being measured, but so far has largely been extrapolated from the data of phylogenetically related viruses. Antibody responses to SARS-CoV-1 persist for two to three years [11] and memory T cells persist for 11 years after infection [12]. In contrast, beta coronaviruses [β-CoV] that are phylogenetically close to SARS-CoV-2 are known to re-infect humans throughout life [13], suggesting shortlasting protective immunity. Human controlled infection models using common cold associated beta coronaviruses (β-CoV) showed partial protection from antibodies that persist for one year [14]. These findings suggest that similar protective immune mechanisms could be operative in SARS-CoV-2 as well but would need detailed characterization. Further, uninfected individuals could harbor antibodies and memory T cells to other beta coronaviruses [15]. Such cross-reactive T cell responses [15] targeting several epitopes on the surface proteins of SARS-CoV-2 [16], could potentially influence the course of infection, or the clinical outcomes. The limited availability of data on SARS-CoV2 infections in LMICs where exposure to other coronaviruses may differ warrant a detailed evaluation of cross-reactive T cell and antibody landscapes in primary infections and re-infection outcomes in the community.

This protocol describes a study to estimate the incidence of infection, re-infection and vaccine breakthrough infections in a community in India. The study would also determine the

For peer review only - http://bmjopen.bmj.com/site/about/guidelines.xhtml

antibody profile, duration of antibody persistence as well the cellular immune responses following natural COVID-19 infection and re-infection.

Objectives and Outcome:

Objective 1: To estimate the seroprevalence of antibodies to SARS-CoV-2 spike protein in Vellore
Outcome:<br>a. The proportion of individuals ≥18 years of age who are seropositive for antibodies to spike protein of SARS-CoV-2 in Vellore<br>b. Prevalence of seropositivity across clusters (wards)
Objective 2: To measure the incidence of SARS-CoV-2 infection in a cohort of individuals >= 18 years in Vellore
Outcome:<br>a. Incidence of SARS-CoV-2 infection among those without evidence of prior infection or vaccination<br>b. Incidence of SARS-CoV-2 infection among those with evidence of prior SARS-CoV-2 infection<br>c. Incidence of SARS-CoV-2 infection in those who have received at least one dose of COVID-19 vaccine
Objective 3: To track cellular and humoral immune correlates of COVID-19 infection, re-infection, and clinically significant disease
Outcome:<br>a. Kinetics and longevity of antibody responses and immunological memory

For peer review only

b. Influence of baseline memory T and B levels (both SARS-CoV-2 specific as well as cross-reactive) on infections

Methods

Study setting: Description of the site

Vellore is a tier 2 city in northern Tamil Nadu with a population of close to 5,00,000. It is divided into four zones and 60 administrative wards. The Vellore Health and Demographic Surveillance System (VHDSS), established by the Christian Medical College, monitors a population of 1,20,000 people across zones 3 and 4 of the city. This study area has a very high population density predominantly belonging to the economically poorer section, and is largely homogenous, with daily wage-earners being the largest sub-group of the population.

Study design

The study will have three components (1) serosurvey to estimate the seroprevalence of SARS-CoV-2 spike protein antibodies in the study area, (2) prospective weekly follow-up to estimate the infection and re-infection rates in a cohort of 1200 individuals, (3) intensive follow up of incident SARS-COV-2 infections (both symptomatic and asymptomatic) to characterize immunological and clinical features of infection in the cohort. The study flow chart is depicted in Figure 1.

Patient and Public involvement

No patients or public involvement in the design or conduct or reporting or dissemination plans of our research.

Inclusion and exclusion criteria

Serosurvey

Inclusion criteria:

1. Above the age of 18 years

For peer review only

2. Permanent residents of the selected wards
3. Only one member from each selected household will be enrolled

Exclusion criteria:

1. Participant refusal of consent
2. Pregnant women and immunocompromised patients will be excluded
3. Acute febrile illness in the participant at the time of the survey

<u>**Longitudinal study**</u>

Inclusion criteria:

1. Individuals with a history of clinical illness suggestive of COVID-19 or confirmed COVID-19 in the past, who are seropositive at baseline in the serosurvey (symptomatic seropositive).
2. Individuals seropositive at baseline, with no history of COVID-19 (asymptomatic seropositive).
3. Individuals seronegative at baseline, stratified by the ward of residence.

Exclusion criteria:

1. Participants who are not willing for intensive follow-up till the end of the study.
2. Participants with immunodeficiency states such as people living with HIV infection.
3. Active cancers or bleeding disorders

<u>**Statistical Consideration.**</u>

Assumptions

We make the following assumptions.

1. 50% of the population will be seropositive at baseline.
2. 40% will have received two doses of vaccine mid-way into the study.
3. The annual incidence of SARS-CoV-2 infection detected by the salivary PCR in those **unvaccinated and have no detectable antibodies** (unexposed) at baseline will be **12%.**

For peer review only

4. The annual incidence of SARS-CoV-2 infection detected by the salivary PCR in those **vaccinated and** unexposed at baseline will be **6%** (VE 50% against infection).
5. The annual incidence of SARS-CoV-2 infection detected by the salivary PCR in those **unvaccinated and who have antibodies (exposed)** at baseline will be **4.8%.**
6. The annual incidence of SARS-CoV-2 infection detected by the salivary PCR in those **vaccinated and exposed** at baseline will be **4%.**

Based on these assumptions, for 90% power to detect a 5% difference in the rate of re-infection and primary infection in the cohort, a sample size of 1200 participants is proposed, assuming a 10% dropout rate.

Key definitions

Seropositive is defined as serum/plasma samples positive for IgG spike protein antibody to SARS CoV2 identified by Chemiluminescence Immunoassay (CLIA) using DiaSorin's Liaison XL.

Past asymptomatic infection refers to those who are seropositive (or documented RT-PCR positive >1 month in the past) but are neither antigen or RTPCR positive at baseline assessment AND have had no symptoms of COVID-19.

Recent asymptomatic infection refers to those who are seronegative AND are either RTPCR or antigen positive AND have had no symptoms of COVID-19.

Past symptomatic infection refers to those who are seropositive (or documented RTPCR positive >1 month in the past) but are neither antigen or RTPCR positive at assessment AND have had symptoms of COVID19 in the past.

Recent symptomatic infection refers to those who are seronegative AND are either RTPCR or antigen positive at assessment AND have symptoms of COVID19 within the past one month.

For peer review only - http://bmjopen.bmj.com/site/about/guidelines.xhtml

Study procedures

Baseline serology screening

A baseline serosurvey, conducted on 2000 individuals in four urban clusters, is planned based on population proportionate to size (PPS). The participants who satisfy the inclusion criteria will be selected for the serosurvey from areas within the Vellore corporation limits after obtaining written informed consent. The inclusion and exclusion criteria are detailed in the earlier section. The baseline demographic information, along with details of any clinically relevant illness in the past one month, will be documented. History of confirmed COVID-19 or COVID-like illnesses during the period of the pandemic will also be documented. A peripheral blood sample (5 ml serum) will be collected.

Establishment of the cohort

Based on the seroprevalence from the serosurvey, longitudinal follow-up will be initiated in the Vellore health and demographic surveillance system (VHDSS) area. A total of 1200 residents living in the densely populated wards of zone 3 and 4 of the Vellore corporation will be recruited for the longitudinal follow-up. Those subjects who agree to the specific terms of the longitudinal follow-up of 24 months will be recruited after informed consent. Each study participant will be assigned a unique cohort ID used for reference during the follow-up period. Upon recruitment, blood samples (15-30 ml) will be collected and stored appropriately. Peripheral Blood Mononuclear Cells (PBMCs) will be isolated prior to storage to assess the baseline T-cell and memory B cell profiles in the future.

For peer review only - http://bmjopen.bmj.com/site/about/guidelines.xhtml

Weekly follow up

An assigned field research assistant (FRA) will contact the study participant every week, either by telephonic or direct visit and collects information regarding any COVID-like symptoms in the preceding week. The study participants will be trained to collect 2 ml of saliva in the universal sample container, early in the morning, on one designated day of the week. The participants will be asked to collect these samples as per the study protocol, prior to routine oral hygiene, and consumption of any food or drink. The samples will be transported to the lab in vaccine carriers with ice packs to maintain a temperature of 4°C. The samples once received in the lab will be aliquoted in two different vials. One vial will be retained at the Wellcome Trust Research Laboratory, Vellore. The other vial is sent to the National Centre for Biological Sciences, Bangalore (NCBS) for RT-PCR.

If an individual tests positive for SARS-CoV2, the weekly salivary sample collection will be suspended for the next 90 days. The weekly contact, however, will be continued. The study participants will be requested to inform the study team if they experience any clinically significant febrile or respiratory distress. Symptomatic individuals will be advised to visit Christian Medical College Hospital, Vellore and get tested by nasopharyngeal RT-PCR, as deemed necessary, after clinical examination.

During the second year of the study, weekly follow up would be through telephonic interviews. Weekly salivary samples would not be collected, and home visits would be done for subjects with symptoms. Any incident infection will be followed up for detailed immunological testing. Once every six months, a blood sample (5 ml) will be collected for assessing the serostatus of the participants to identify any infection that was missed through the RT-PCR screening. Sequencing will be done on all positive samples to identify the genetic sequence of the virus at NCBS, Bangalore.

For peer review only

Detailed follow-up of COVID-19 infections

All COVID-19 infections, including symptomatic and asymptomatic will be followed up from the day of the positive report (Day 0). Blood samples (30ml) are collected for PBMC isolation and storage within 24 hours of identification of positives on (Day-0), Day-30 (+2 days) and Day-90 (+7 days) post-infection.

Sample collection

Blood sample -serology*:* Five ml of peripheral blood will be collected (in serum tubes) from 2000 individuals during the baseline serosurvey, and once every six months from the 1200 study participants who are a part of the longitudinal cohort.

Salivary sample*:* Salivary samples will be self-collected, stored and transported to the NCBS laboratory, Bengaluru, as per the Standard Operating Procedure, once a week during the first year of the study. The results will be uploaded timely into the secure data entry portal designed for the laboratory.

Nasopharyngeal swab*:* If any study participants report any clinically significant febrile illness or respiratory distress, they will be offered a medical consultation, and when necessary, a nasopharyngeal RT-PCR at CMC or in any institute of their choice.

Blood sample (for PBMC): 30 ml (minimum 15 mL) of blood will be collected (in 9 ml heparin tubes) after recruitment into the longitudinal study and for confirmed SARS-CoV-2 infections on Day-0, Day-30 and Day-90. PBMCs will be separated by density gradient centrifugation method and cryopreserved in liquid nitrogen.

Laboratory procedures

Weekly salivary samples

Upon receipt and aliquoting, salivary samples will be pooled for testing on the same day. Ten µl of five samples each will be pooled in a single well of the PCR plate, and 6µl of proteinase

For peer review only - http://bmjopen.bmj.com/site/about/guidelines.xhtml

K of 50 µg/µl concentration will be added to each well. Subsequently, the plates will be sealed and heated at 95° Celsius for 5 minutes in a dry thermal bath. After heat inactivation, the plates will be stored at minus 80°C. The pooled PCR plate and an aliquot of saliva will be transported on dry ice to NCBS. RT-PCR will be performed on the pooled samples targeting the N gene, E gene and RdRp gene of SARS CoV 2. If any pool turns out to be positive, RT-PCR will be performed on individual samples. All positive samples will undergo sequencing.

<u>Blood samples</u>

Serological assays

The plasma or serum sample collected at different time points will be tested for IgG antibody against spike protein using a high throughput automated platform. (Diasorin LiaisonXL)

Immunophenotyping

Quantitation of SARS-CoV-2 specific T cells will be done by flow cytometric detection of cytokines and Activation Induced Marker (AIM) upregulation in T cells after stimulation with peptide pools. PBMC stimulation will be done using a 10-mer peptide pool for CD8 and 20-mer peptide-pools for CD4 T cells. Four peptide pools will be used, corresponding to the major proteins of SARS-CoV-2 (Spike, Envelope, Membrane and Nucleoprotein). For all the stimulation conditions, one well (vehicle-treated) will act as negative control. An additional well of cytomegalovirus (CMV)-peptide-stimulated control (a mix of 10-mer and 15-mer CMV peptides) will be kept as positive control for each sample. Baseline levels of cross-reactive T cells to non-SARS-CoV-2 human Coronaviruses (hCoV) will be estimated using the same methodology, using peptide pools derived from hCoV strains. Memory B cells will be detected by flow cytometry after staining PBMCs with fluorophore-tagged viral proteins and memory B cell markers.

For peer review only

312 **Statistical Analysis**

313 Seroprevalence is estimated as a proportion and will be assumed to follow a binomial
314 distribution. The incidence of infection within the cohort is expected to follow a Poisson
315 distribution. We will permit repeated infections to be captured in analysis and account for the
316 same in the analysis. A time to event analysis using Prentice, Williams and Peterson models
317 comparing incidence in the exposed and unexposed cohorts will be performed. We will adjust
318 for background infection rates in each cluster (ward) and covariates such as age, SES,
319 vaccination status, per-capita floor space and occupation class.

320 The statistical analysis plan will detail the estimation of seroprevalence, its risk factors, the
321 incidence of primary and re-infection and a comparison of these rates.

322 **Key comparisons in the study**

323 We will make comparisons between

324 - Incidence rates of infection overall and in seropositive and seronegative subgroups.
325 - Incidence rates of infection among the vaccinated individuals in the cohorts
326 - Kinetics and longevity of memory B and T cells in infections occurring in the
327 seropositive and seronegative cohort
328 - Baseline cross-reactive T cells and antibodies to non-SARS-CoV-2 beta coronaviruses
329 between symptomatic infections vs asymptomatic infections vs uninfected individuals
330 in the seronegative cohort
331 - Baseline SARS-CoV-2 specific memory T and B cells and antibody levels between
332 infected individuals versus uninfected individuals in the seropositive cohort

333 **Data Management Plan**

334 All the Case Report Format (CRFs) will be in the electronic format (Redcap©), and the entry
335 platform will be connected to the Central database server. The Data management system is
336 responsible for the periodic validation process and quality of the data. Any further correction

For peer review only - http://bmjopen.bmj.com/site/about/guidelines.xhtml
For peer review only

in the database after the entry is 'saved' is accompanied by a duly completed "Data Clarification form." The electronic data management system tracks key study progress parameters on an access-restricted online dashboard. The weekly contact made by the field research assistants will be independently validated by a field worker who calls 5% of all individuals who were contacted that week.

Discussion

To our knowledge, this study is the first to follow up a cohort in an LMIC, for a period of two years for COVID-19 infection and re-infection. In terms of surveillance of SARS-CoV-2 infection, though the nasopharyngeal swab has been the gold standard for diagnosis, the use of saliva samples will be an acceptable alternate by the study participants as it is self-directed, non-invasive and has a good concordance with the nasopharyngeal swab. The study aims to address several gaps in the current scientific evidence of SARS-CoV-2 infection and immunity. Firstly, there are a limited number of studies that investigate the long term follow up of individuals for the rates of infection and re-infection in the community. Secondly, the study aims to look at the kinetics of IgG antibodies following infection. The cross-reactivity between SARS-CoV-2 and other human coronaviruses will support better understanding of determinants of symptomatic infection. The T cell and B cell memory responses would help in understanding the kinetics and longevity of immune responses in seropositive and seronegative individuals and would help in decision making with regard to booster vaccination.

To conclude, CORES will help in estimating the re-infection rates, detailed immunogenicity amongst the COVID-19 positive individuals, establish the antibody kinetics and characterise the breakthrough infections amongst the vaccinated individuals in the community.

For peer review only

For peer review only - http://bmjopen.bmj.com/site/about/guidelines.xhtml

Statements

a. Contributorship statement: The study design and concept were conceived by JJ and GK. RM will conduct the study as part of her PhD under the supervision of GK, SB, SP and JJ. JSP and JJ designed the process evaluation and wrote the statistical analysis plan and JJ, DK and JSP organise data management and will oversee field operations. All authors provided edits and critiqued the manuscript for the scientific content. All authors read and approved the final version of the manuscript.

b. Competing interests: The authors declare that they have no competing interests.

c. Funding: This study is funded by Bill and Melinda Gates Foundation funding INV-024915. The funders will have no role in the design of this study and will not have any role during its execution, analyses, interpretation of the data, or decision to submit results.

d. Data sharing statement: No data are available.

References:

1. Poland GA, Ovsyannikova IG, Kennedy RB. SARS-CoV-2 immunity: review and applications to phase 3 vaccine candidates. The Lancet. 2020 13 October.
2. Okba NM, Müller MA, Li W, Wang C, GeurtsvanKessel CH, Corman VM, Lamers MM, Sikkema RS, de Bruin E, Chandler FD, Yazdanpanah Y. Severe acute respiratory syndrome coronavirus 2− specific antibody responses in coronavirus disease patients. Emerging infectious diseases. 2020 Jul;26(7):1478-88.
3. Chen X, Pan Z, Yue S, Yu F, Zhang J, Yang Y, Li R, Liu B, Yang X, Gao L, Li Z. Disease severity dictates SARS-CoV-2-specific neutralizing antibody responses in COVID-19. Signal transduction and targeted therapy. 2020 Sep 2;5(1):1-6.
4. Tan W, Lu Y, Zhang J, Wang J, Dan Y, Tan Z, He X, Qian C, Sun Q, Hu Q, Liu H. Viral kinetics, and antibody responses in patients with COVID-19. MedRxiv. 2020 1 January.

For peer review only

For peer review only - http://bmjopen.bmj.com/site/about/guidelines.xhtml

5. Huang J, Mao T, Li S, Wu L, Xu X, Li H, Su F, Dai J, Shi J, Cai J, Huang C. Long period dynamics of viral load and antibodies for SARS-CoV-2 infection: an observational cohort study. medRxiv. 2020 1 January.

6. Moderbacher CR, Ramirez SI, Dan JM, Grifoni A, Hastie KM, Weiskopf D, Belanger S, Abbott RK, Kim C, Choi J, Kato Y. Antigen-specific adaptive immunity to SARS-CoV-2 in acute COVID-19 and associations with age and disease severity. Cell. 2020 16 September.

7. To KK, Hung IF, Ip JD, Chu AW, Chan WM, Tam AR, Fong CH, Yuan S, Tsoi HW, Ng AC, Lee LL. COVID-19 re-infection by a phylogenetically distinct SARS-coronavirus-2 strain confirmed by whole genome sequencing. Clinical Infectious Diseases. 2020 25 August.

8. Tillett RL, Sevinsky JR, Hartley PD, Kerwin H, Crawford N, Gorzalski A, Laverdure C, Verma SC, Rossetto CC, Jackson D, Farrell MJ. Genomic evidence for re-infection with SARS-CoV-2: a case study. The Lancet Infectious Diseases. 2020 12 October.

9. Bylicki O, Delarbre D, Mayet A, Ferrier A, Perisse A, Malle C, et al. Neutralizing antibody response to SARS-CoV-2 persists 9 months post symptom onset in mild and asymptomatic patients. Int J Infect Dis. 2021;112:8–12.

10. Zhu L, Xu X, Zhu B, Guo X, Xu K, Song C, et al. Kinetics of SARS-CoV-2 Specific and Neutralizing Antibodies over Seven Months after Symptom Onset in COVID-19 Patients. Microbiol Spectr. 2021;9:e0059021.

11. Cao WC, Liu W, Zhang PH, Zhang F, Richardus JH. Disappearance of antibodies to SARS-associated coronavirus after recovery. New England Journal of Medicine. 2007 Sep 13;357(11):1162-3.

For peer review only - http://bmjopen.bmj.com/site/about/guidelines.xhtml

12. Ng OW, Chia A, Tan AT, Jadi RS, Leong HN, Bertoletti A, Tan YJ. Memory T cell responses targeting the SARS coronavirus persist up to 11 years post-infection. Vaccine. 2016 Apr 12;34(17):2008-14.

13. Gaunt ER, Hardie A, Claas EC, Simmonds P, Templeton KE. Epidemiology and clinical presentations of the four human coronaviruses 229E, HKU1, NL63, and OC43 detected over 3 years using a novel multiplex real-time PCR method. Journal of clinical microbiology. 2010 1 August;48(8):2940-7.

14. Callow KA, Parry HF, Sergeant M, Tyrrell DA. The time course of the immune response to experimental coronavirus infection of man. Epidemiology & Infection. 1990 Oct;105(2):435-46.

15. Braun J, Loyal L, Frentsch M, Wendisch D, Georg P, Kurth F, Hippenstiel S, Dingeldey M, Kruse B, Fauchere F, Baysal E. SARS-CoV-2-reactive T cells in healthy donors and patients with COVID-19. Nature. 2020 Jul 29:1-5.

16. Mateus J, Grifoni A, Tarke A, Sidney J, Ramirez SI, Dan JM, Burger ZC, Rawlings SA, Smith DM, Phillips E, Mallal S. Selective and cross-reactive SARS-CoV-2 T cell epitopes in unexposed humans. Science. 2020 2 October;370(6512):89-94.

Abbreviations:

CAP	Chinnallapuram
CMC	Christian Medical College, Vellore
CMV	Cytomegalovirus
COVID-19	Coronavirus Disease-2019
CRF	Case Report Format
FRA	Field Research Assistant
hCoV	Human Coronavirus
LMIC	Low and Middle Income Country

For peer review only

NCBS	National Centre for Biological Sciences
PBMC	Peripheral Blood Mononuclear Cells
PPS	Probability proportionate to size
RT-PCR	Reverse Transcription -Polymerase Chain Reaction
SARS-COV-2	Severe acute respiratory syndrome coronavirus 2
SES	Socio Economic Status
ß-CoV	Betacoronavirus
VHDSS	Vellore Health & Demographic Surveillance System

Ethics approval and consent to participate:

The study has been approved by the institutional review board, (IRB No: 13585), Christian Medical College, Vellore. The study will adhere to the principles that govern biomedical research involving human subjects. The Declaration of Helsinki will be followed to assure that the rights, integrity, and confidentiality of study participants are protected, and that reported results are credible and accurate. The privacy and confidentiality of all information collected, including those derived from clinical specimens, will be ensured during and after the project. Individuals will not be identified in any reports or publications based on the study. All participant data will be computerized using password protection. The participants will be asked to provide written informed consent.

Consent for publication: Not applicable.

Acknowledgements: We would like to acknowledge the participants who are willing to participate in the current study and help us in understanding the current knowledge gaps in COVID-19 infection and re-infections.

For peer review only - http://bmjopen.bmj.com/site/about/guidelines.xhtml

We would like to thank the National Centre for Biological Sciences, Bangalore for helping us in processing our weekly saliva samples.

Figure legend

Figure 1: CORES study flowchart

For peer review only

For peer review only - http://bmjopen.bmj.com/site/about/guidelines.xhtml

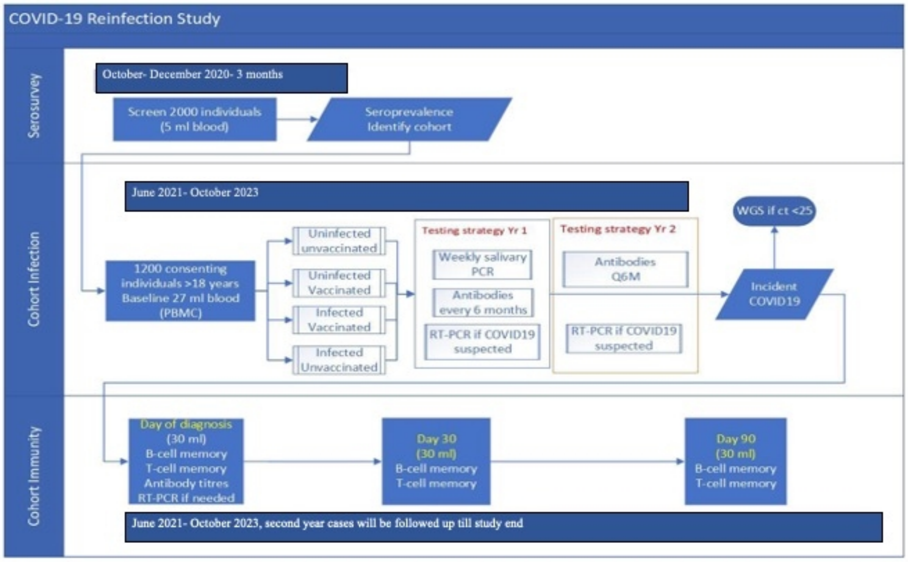

CORES study flowchart

338x190mm (54 x 54 DPI)

BMJ Open

Risk of COVID-19 re-infection and its predictors (CORES): protocol for a community based longitudinal cohort study in Vellore, India

Journal:	*BMJ Open*
Manuscript ID	bmjopen-2021-059869.R1
Article Type:	Protocol
Date Submitted by the Author:	20-Apr-2022
Complete List of Authors:	Madhavan, Ramya; Christian Medical College Vellore, The Wellcome Trust Research Laboratory<br>Paul, Jackwin; Christian Medical College Department of Community Health and Development<br>Babji, Sudhir; Christian Medical College and Hospital Vellore<br>Kumar, Dilesh; Christian Medical College Vellore<br>Prabhu, Savit; Christian Medical College and Hospital Vellore, Department of GI Sciences<br>Pulleri, Harsha; inStem<br>Annadorai, Ravikiran; inStem<br>Gowda, Sampreeth; inStem<br>John, Jacob; Christian Medical College and Hospital Vellore, Department of Community Health and Development<br>Kang, Gagandeep; Christian Medical College Vellore, Department of GI Sciences
**Primary Subject Heading**:	Public health
Secondary Subject Heading:	Epidemiology, Infectious diseases
Keywords:	COVID-19, IMMUNOLOGY, PUBLIC HEALTH

SCHOLARONE™ Manuscripts

Risk of COVID-19 re-infection and its predictors (CORES): protocol for a community based longitudinal cohort study in Vellore, India

Joint First Authors:

Ramya Madhavan*

Research Fellow

The Wellcome Trust Research laboratory

Division of GI Sciences

Christian Medical College, Vellore

India

E-mail: ramya.m@cmcvellore.ac.in

Jackwin Sam Paul*

Assistant Professor

Department of Community Health

Christian Medical College, Vellore

India

E-mail: jackwin.sam@cmcvellore.ac.in

*- Both authors contributed equally and would be joint first authors

Co-authors:

Sudhir Babji

Professor

The Wellcome Trust Research laboratory

Division of GI Sciences

Christian Medical College, Vellore

For peer review only

India

E-mail: sudhirbabji@cmcvellore.ac.in

Dilesh Kumar. A

Research Officer

The Wellcome Trust Research laboratory

Division of GI Sciences

Christian Medical College, Vellore

India.

E-mail: dilesh.kumara@cmcvellore.ac.in

Savit B Prabhu

Assistant Professor

The Wellcome Trust Research laboratory

Division of GI Sciences

Christian Medical College, Vellore

India.

E-mail: savit.b@cmcvellore.ac.in

Pulleri Kandi Harsha

Project Scientist II

Institute of Stem Cell science and Regenerative Medicine

Bangalore,

India

E-mail: harshapk@instem.res.in

Ravikiran Annadorai

Project Associate

Institute of Stem Cell science and Regenerative Medicine

For peer review only

Bangalore,

India

E-mail: ravikirana@instem.res.in

Gowda Sampreeth Ravi

Project Associate

Institute of Stem Cell science and Regenerative Medicine

Bangalore,

India

E-mail: sampreethg@instem.res.in

Jacob John

Professor

Department of Community Health

Christian Medical College, Vellore

E-mail: jacob@cmcsph.org

Correspondence to:

Gagandeep Kang

Professor of Microbiology

The Wellcome Trust Research laboratory

Division of GI Sciences

Christian Medical College, Vellore

India

E-mail: gkang@cmcvellore.ac.in

For peer review only

For peer review only - http://bmjopen.bmj.com/site/about/guidelines.xhtml

Abstract

Introduction

The incidence of SARS-CoV-2 re-infection has not been widely evaluated in low-income and middle-income countries (LMICs). Understanding immune responses elicited by SARS-CoV-2 natural infection and factors that lead to re-infection in a community setting is important for public health policy. We aim to investigate the risk of primary infection and re-infection among those without and with evidence of prior infection as defined by the presence of antibodies to SARS-CoV-2 spike protein.

Methods and analysis

A baseline seroprevalence survey will test for SARS-CoV2 antibodies among healthy adults in Vellore, India. Based on an expected seropositivity rate of 50% in the general population, with an annual attack rate of 12%, 6%, 4.8% and 4% among those unvaccinated and seronegative, vaccinated and seronegative, unvaccinated and seropositive, and vaccinated and seropositive respectively, we will recruit 1200 adults who will be followed up for a total of 24 months. Weekly self-collected saliva samples will be tested by RT-PCR to detect SARS-CoV2 infections, for a period of one year. For any person testing RT-PCR positive, blood samples will be collected within 2 days of RT-PCR positivity and on days 30 and 90 to assess the kinetics and longevity of the antibody responses, B cell memory and T cell memory post-infection. The data will be analyzed to estimate seroprevalence at baseline and over time, the risk factors for infection, rates of primary infection and re-infection and provide a comparison of the rates across groups based on infection and vaccination status.

For peer review only

For peer review only - http://bmjopen.bmj.com/site/about/guidelines.xhtml

Ethics and dissemination

The study has been approved by the institutional review board (IRB No: 13585), Christian Medical College, Vellore. The results of the study will be made available through journal publications and conference presentations.

Study registration

The study has been registered with the Central Trial Registry of India (CTRI; registration number CTRI/2020/11/029438).

Strengths and limitations of this study

- The use of saliva samples for SARS CoV 2 surveillance will be an acceptable alternate as it is self-directed and non-invasive.
- Weekly salivary RT-PCR will serve as surveillance for SARS CoV 2 at the community level in Vellore.
- The study involves analysis of both humoral and cellular immune responses in individuals with infections and re-infections.
- The immunological profile following vaccine breakthrough infections will be studied in detail.
- Though there is a good concordance of saliva and nasopharyngeal swab for SARS CoV 2 surveillance, there could be some infections which may be missed with saliva sampling.

Keywords: COVID-19, Immunology, Public Health.

For peer review only - http://bmjopen.bmj.com/site/about/guidelines.xhtml

Introduction

Immune responses to SARS-CoV-2 infection, vaccination, and the immune correlates of protection are areas of active investigation [1–4]. A few studies have shown that the development, amount, kinetics of antibodies may correlate with the clinical outcome of SARS CoV 2 infections. [5–7]. The coordinated response between humoral and cellular immunity has been hypothesized to be protective [8]. From a public health perspective, it is crucial to understand the duration of protective immunity offered by natural infections and vaccination. The reported duration of protection following a natural infection is around 8 months to 1 year. [2–4]. Re-infections from a different strain have been documented in persons who have recovered from a prior natural infection.[9,10] At the population level, the incidence of re-infection over a longer term of one to two years due to various variants of concern (VOC) has not been evaluated, and this is also affected by vaccination. Preliminary studies suggest that antibodies persist for seven to nine months or more post-infection [11,12]. The rates of attrition of potential immune correlates like memory B and T cell responses, and the association of these humoral and cellular immune parameters with subsequent re-infections, particularly with VOCs are unknown. The duration of protective immunity to SARS-CoV-2 is being measured, but so far has largely been extrapolated from the data of phylogenetically related viruses. Antibody responses to SARS-CoV-1 persist for two to three years [13] and memory T cells persist for 11 years after infection [14]. In contrast, beta coronaviruses [β-CoV] that are phylogenetically close to SARS-CoV-2 are known to re-infect humans throughout life [15], suggesting short lasting protective immunity. Human controlled infection models using common cold associated beta coronaviruses (β-CoV) showed partial protection from antibodies that persist for one year [16]. These findings suggest that similar protective immune mechanisms could be operative in SARS-CoV-2 as well but need detailed characterization in populations with known viral circulation. Further, uninfected individuals could harbor antibodies and memory T cells to other

For peer review only

beta coronaviruses [17]. Such cross-reactive T cell responses [17] targeting several epitopes on the surface proteins of SARS-CoV-2, could potentially influence the course of infection, or the clinical outcomes. The limited availability of data on SARS-CoV2 infections in LMICs where exposure to other coronaviruses may differ, warrant a detailed evaluation of cross-reactive T cell and antibody landscapes in primary infections and re-infection outcomes in the community.

This protocol describes a study to estimate the incidence of infection, re-infection and vaccine breakthrough infections in a community in India. The study would also determine the antibody profile, duration of antibody persistence as well the cellular immune responses following natural COVID-19 infection and re-infection.

Objectives and Expected outcomes

The CORES study has the objectives and outcomes as described in table 1.

Table 1: Objectives and outcomes of CORES study

Objective 1: To estimate the seroprevalence of antibodies to SARS-CoV-2 spike protein in Vellore (May- October 2021)
Outcome:<br>a. The proportion of individuals ≥18 years of age who are seropositive for antibodies to spike protein of SARS-CoV-2 in Vellore<br>b. Prevalence of seropositivity across clusters (wards)
Objective 2: To measure the incidence of SARS-CoV-2 infection in a cohort of individuals >= 18 years in Vellore (May 2021- October 2023)
Outcome:<br>a. Incidence of SARS-CoV-2 infection among those without evidence of prior infection or vaccination

For peer review only

b. Incidence of SARS-CoV-2 infection among those with evidence of prior SARS-CoV-2 infection
c. Incidence of SARS-CoV-2 infection in those who have received at least one dose of COVID-19 vaccine at least 14 days prior to infection.

Objective 3: To track cellular and humoral immune correlates of COVID-19 infection, re-infection, and clinically significant disease (May 2021- October 2023)

Outcome:

a. Kinetics and longevity of antibody responses and immunological memory
b. Influence of baseline memory T and B levels (both SARS-CoV-2 specific as well as cross-reactive) on infection

For peer review only

Methods and analysis

Study setting

Vellore is a tier 2 city in northern Tamil Nadu with a population of close to 5,00,000. It is divided into four zones and 60 administrative wards. The Vellore Health and Demographic Surveillance System (VHDSS), established by the Christian Medical College, monitors a population of 1,20,000 people across zones 3 and 4 of the city. This study area has a very high population density predominantly belonging to the economically poorer section, and is largely homogenous, with daily wage-earners being the largest sub-group of the population.

Study design

The study will have three components (1) serosurvey to estimate the seroprevalence of SARS-CoV-2 spike protein antibodies in the study area (2) prospective weekly follow-up to estimate the infection and re-infection rates in a cohort of 1200 individuals, (3) intensive follow up of incident SARS-COV-2 infections (both symptomatic and asymptomatic) to characterize immunological and clinical features of infection in the cohort. The study flow is in Figure 1.

For peer review only - http://bmjopen.bmj.com/site/about/guidelines.xhtml

Study status and timeline

The cohort recruitment started on 11th May 2021 and was completed on 28th October 2021. The cohort will be followed up for a period of two years and data will be collected until October 2023.

Patient and public involvement

No patients or public were involved in the design or conduct of the study. We will report the data in peer reviewed publications and share it with state health authorities. Participants will be provided with study results and interpretation at a public meeting at the end of the study.

Inclusion and exclusion criteria

Inclusion criteria:

1. Age 18 years and above
2. Permanent residents of the selected wards
3. Only one member from each selected household will be enrolled
4. Individuals with a history of clinical illness suggestive of COVID-19 or confirmed COVID-19 in the past, who are seropositive at baseline in the serosurvey (symptomatic seropositive).
5. Individuals seropositive at baseline, with no history of COVID-19 (asymptomatic seropositive).
6. Individuals seronegative at baseline, stratified by the ward of residence.

Exclusion criteria:

1. Participant refusal of consent
2. Pregnant women and immunocompromised patients
3. Participants not willing for follow-up till the end of the study.
4. Active cancers or bleeding disorders.

Statistical considerations

Assumptions

For peer review only - http://bmjopen.bmj.com/site/about/guidelines.xhtml

We make the following assumptions. The assumptions were based on the early findings of the Com-CoV study as there were no published data regarding vaccine efficacy and re-infections prior to the start of the study.[18]

1. 50% of the population will be seropositive at baseline.
2. 40% will have received two doses of vaccine mid-way into the study.
3. The annual incidence of SARS-CoV-2 infection detected by the salivary PCR in those **unvaccinated and have no detectable antibodies** (unexposed) at baseline will be **12%.**
4. The annual incidence of SARS-CoV-2 infection detected by the salivary PCR in those **vaccinated and** unexposed at baseline will be **6%** (VE 50% against infection).
5. The annual incidence of SARS-CoV-2 infection detected by the salivary PCR in those **unvaccinated and who have antibodies (exposed)** at baseline will be **4.8%.**
6. The annual incidence of SARS-CoV-2 infection detected by the salivary PCR in those **vaccinated and exposed** at baseline will be **4%.**

Based on these assumptions, for 90% power to detect a 5% difference in the rate of re-infection and primary infection in the cohort, a sample size of 1200 participants is proposed, after allowing for a 10% dropout rate.

Key definitions

Seropositive is defined as serum/plasma samples positive for IgG spike protein antibody to SARS CoV2 identified by LIAISON® SARS-CoV-2 TrimericS IgG assay by Diasorin platform. The cut off for seropositivity is more than or equal to 33.8 BAU/ml.

Past asymptomatic infection refers to those who are seropositive (or documented RT-PCR positive >1 month in the past) but are neither antigen or RTPCR positive at baseline assessment AND have had no symptoms of COVID-19.

For peer review only - http://bmjopen.bmj.com/site/about/guidelines.xhtml

Recent asymptomatic infection refers to those who are seronegative AND are either RTPCR or antigen positive AND have had no symptoms of COVID-19.

Past symptomatic infection refers to those who are seropositive (or documented RTPCR positive >1 month in the past) but are neither antigen or RTPCR positive at assessment AND have had symptoms of COVID19 in the past.

Recent symptomatic infection refers to those who are seronegative AND are either RTPCR or antigen positive at assessment AND have symptoms of COVID19 within the past one month.

Clinically significant disease refers to those who develop symptoms due to SARS CoV 2 and require hospitalisation or Intensive Care Unit admission.

Re-positivity refers to those who test positive within 90 days of the first RT-PCR results with symptoms.

Re-infection refers to those who test positive after 90 days of the first RT-PCR results with or without any symptoms.

For peer review only

Study procedures

Baseline serology screening

A baseline serosurvey, conducted on 2000 individuals in four urban clusters, is planned based on population proportionate to size (PPS). The participants who satisfy the inclusion criteria will be selected for the serosurvey from areas within the Vellore corporation limits after obtaining written informed consent. The inclusion and exclusion criteria are detailed in the earlier section. The baseline demographic information, along with details of any clinically relevant illness in the past one month, will be documented. History of confirmed COVID-19 or COVID-like illnesses during the period of the pandemic will also be documented. A peripheral blood sample (5 ml serum) will be collected.

For peer review only - http://bmjopen.bmj.com/site/about/guidelines.xhtml

Establishment of the cohort

Based on the seroprevalence from the serosurvey, longitudinal follow-up will be initiated in the Vellore health and demographic surveillance system (VHDSS) area. A total of 1200 residents living in the densely populated wards of zone 3 and 4 of the Vellore corporation will be recruited for the longitudinal follow-up. Those subjects who agree to the specific terms of the longitudinal follow-up of 24 months will be recruited after informed consent. One member in the household will be selected using simple random sampling. Each study participant will be assigned a unique cohort ID. The final 1200 participants will be in any of the four groups based on their vaccination and infection status with no specific distribution across these four groups. The vaccination status will be obtained and recorded at the baseline and every 6 months for those who were unvaccinated at enrolment. Details of precautionary or booster doses also will be captured during the 6 monthly interview. The vaccination certificate would be verified for confirmation of details (date, type of vaccine, number of doses etc). Upon recruitment, blood samples (15-30 ml) will be collected, processed and stored as per standard protocol. Peripheral Blood Mononuclear Cells (PBMCs) will be isolated prior to storage to assess the baseline T-cell and memory B cell profiles in the future.

Intensive follow up phase

The first year following recruitment of the cohort would be the intensive follow up phase during which weekly follow up visits and saliva sampling is planned. An assigned field research assistant (FRA) will contact the study participant every week, either by telephonic or direct visit and collect information regarding any COVID-like symptoms in the preceding week. The study participants will be trained to collect 2 ml of saliva in the universal sample container, early in the morning, on one designated day of the week. The participants will be asked to collect these samples as per the study protocol, prior to routine oral hygiene, and consumption of any food or drink. The samples will be collected by the FRAs and transported to the lab in

For peer review only - http://bmjopen.bmj.com/site/about/guidelines.xhtml

vaccine carriers with ice packs to maintain a temperature of 4°C. The samples once received in the lab will be aliquoted in two vials. One vial will be retained at the Wellcome Trust Research Laboratory, Vellore. The other vial is sent to the National Centre for Biological Sciences, Bangalore (NCBS) for RT-PCR.

If an individual tests positive for SARS-CoV2, the weekly salivary sample collection will be suspended for the next 90 days. The weekly contact, however, will be continued. During the weekly contact if a subject develops symptoms, their samples will be collected. If they are RT-PCR positive within 90 days it will be considered as re-positive. The study participants will be requested to inform the study team if they experience any clinically significant febrile or respiratory distress. Symptomatic individuals will be advised to visit Christian Medical College Hospital, Vellore and get tested by nasopharyngeal RT-PCR, as deemed necessary, after clinical examination. Clinical symptoms, response to treatment and details of treatment during hospitalization or during home management would be recorded on the Case Report Form (CRF) for every participant who is positive by RT PCR.

Follow up phase - second year

During the second year of the study, weekly follow up would be through telephonic interviews. Weekly salivary samples will not be collected, and home visits will be done only for subjects with symptoms. Any incident infection will be followed up for detailed immunological testing. Once every six months, a blood sample (5 ml) will be collected for assessing the serostatus of the participants to identify any infection that was missed through the RT-PCR screening. Sequencing will be done on all positive samples to identify the genetic sequence of the virus at NCBS, Bangalore and help us determine which variant of concern was responsible for the infections and re-infections. This will include samples classified as 're-positives'.

For peer review only - http://bmjopen.bmj.com/site/about/guidelines.xhtml

297 **Detailed follow-up of COVID-19 infections**

298 All COVID-19 infections, including symptomatic and asymptomatic will be followed up from
299 the day of the positive report (Day 0). Blood samples (30ml) will be collected for PBMC
300 isolation and storage within 24 hours of identification of positives on (Day-0), Day-30 (+2
301 days) and Day-90 (+7 days) post-infection.

302 **Sample collection**

303 **Blood sample - serology**: Five ml of peripheral blood will be collected (in serum tubes) from
304 2000 individuals during the baseline sero-survey, and once every six months from the 1200
305 study participants who are a part of the longitudinal cohort.

306 **Salivary sample***:* Salivary samples will be self-collected, stored and transported to the NCBS
307 laboratory, Bengaluru, as per the Standard Operating Procedure, once a week during the first
308 year of the study. The results will be uploaded into the secure data entry portal designed for
309 the laboratory.

310 **Nasopharyngeal swab***:* If any study participants report any clinically significant febrile illness
311 or respiratory distress, they will be offered a medical consultation, and when necessary, a
312 nasopharyngeal RT-PCR at CMC or in any institute of their choice.

313 **Blood sample (for PBMC):** 30 ml (minimum 15 mL) of blood will be collected (in 9 ml
314 heparin tubes) after recruitment into the longitudinal study and for confirmed SARS-CoV-2
315 infections on Day-0, Day-30 and Day-90. PBMCs will be separated by density gradient
316 centrifugation method and cryopreserved in liquid nitrogen.

317 **Laboratory procedures**

318 **Weekly salivary samples**

319 Upon receipt and aliquoting, salivary samples will be pooled for testing on the same day. Ten
320 µl of five samples each will be pooled in a single well of the PCR plate, and 6µl of proteinase

For peer review only - http://bmjopen.bmj.com/site/about/guidelines.xhtml

K of 50 µg/µl concentration will be added to each well. Subsequently, the plates will be sealed and heated at 95° Celsius for 5 minutes in a dry thermal bath. After heat inactivation, the plates will be stored at minus 80°C. The pooled PCR plate and an aliquot of saliva will be transported on dry ice to NCBS. RT-PCR will be performed on the pooled samples targeting the N gene, E gene and RdRp gene of SARS CoV 2. The limit of detection of the commercial kit that is used for testing is 100 copies/ ml and the sensitivity of detection in saliva samples is around 94% compared to NP swab. [19] If any pool turns out to be positive, RT-PCR will be performed on individual samples. All positive samples will undergo sequencing.

Blood samples

Serological assays

The plasma or serum sample collected at different time points will be tested for IgG antibody against spike protein using a high throughput automated platform (Diasorin LiaisonXL).

Immunophenotyping

Quantitation of SARS-CoV-2 specific T cells will be done by flow cytometric detection of cytokines and Activation Induced Marker (AIM) upregulation in T cells after stimulation with peptide pools. PBMC stimulation will be done using a 10-mer peptide pool for CD8 and 20-mer peptide-pools for CD4 T cells. Four peptide pools will be used, corresponding to the major proteins of SARS-CoV-2 (Spike, Envelope, Membrane and Nucleoprotein). For all the stimulation conditions, one well (vehicle-treated) will act as negative control. An additional well of cytomegalovirus (CMV)-peptide-stimulated control (a mix of 10-mer and 15-mer CMV peptides) will be kept as positive control for each sample. Baseline levels of cross-reactive T cells to non-SARS-CoV-2 human Coronaviruses (hCoV) will be estimated using the same methodology, using peptide pools derived from hCoV strains. Memory B cells will be detected by flow cytometry after staining PBMCs with fluorophore-tagged viral proteins and memory B cell markers.

For peer review only - http://bmjopen.bmj.com/site/about/guidelines.xhtml

Statistical analysis

Seroprevalence is estimated as a proportion and will be assumed to follow a binomial distribution. The incidence of infection within the cohort is expected to follow a Poisson distribution. We will permit repeated infections to be captured in analysis and account for the same in the analysis. A time to event analysis using Prentice, Williams and Peterson models comparing incidence in the exposed and unexposed cohorts will be performed. We will adjust for background infection rates in each cluster (ward) and covariates such as age, SES, vaccination status, per-capita floor space and occupation class.

The statistical analysis plan will detail the estimation of seroprevalence, its risk factors, the incidence of primary and re-infection and a comparison of these rates. Continuous variables will be described using mean (SD) and median (IQR) where necessary. Categorical data will be expressed as frequency (%). Incidence of infection and re-infection will be calculated per thousand person years. Hazard ratios will be estimated to assess protection/risk conferred by vaccination and previous infection.

Key comparisons in the study

We will make comparisons between:

- Incidence rates of infection overall and in seropositive and seronegative subgroups
- Incidence rates of infection among the vaccinated individuals in the cohorts
- Kinetics and longevity of memory B and T cells in infections occurring in the seropositive and seronegative cohort
- Baseline cross-reactive T cells and antibodies to non-SARS-CoV-2 beta coronaviruses between symptomatic infections vs asymptomatic infections vs uninfected individuals in the seronegative cohort
- Baseline SARS-CoV-2 specific memory T and B cells and antibody levels between infected individuals versus uninfected individuals in the seropositive cohort

For peer review only

For peer review only - http://bmjopen.bmj.com/site/about/guidelines.xhtml

Data management plan

All the Case Report Format (CRFs) will be in the electronic format (Redcap©), and the entry platform will be connected to the Central database server. The Data management system is responsible for the periodic validation process and quality of the data. Any further correction in the database after the entry is 'saved' is accompanied by a duly completed "Data Clarification form." The electronic data management system tracks key study progress parameters on an access-restricted online dashboard. The weekly contact made by the FRAs will be independently validated by a field worker who calls 5% of all individuals who were contacted that week.

Ethics and dissemination

The study has been approved by the institutional review board (IRB No: 13585), Christian Medical College, Vellore. The study will adhere to the principles that govern biomedical research involving human subjects as required in India. The Declaration of Helsinki will be followed to assure that the rights, integrity, and confidentiality of study participants are protected, and that reported results are credible and accurate. The privacy and confidentiality of all information collected, including those derived from clinical specimens, will be ensured during and after the project. Individuals will not be identified in any reports or publications based on the study. All participant data will be computerized using password protection. The participants will be asked to provide written informed consent. The knowledge gained and the results will be made available through journal publications and conference presentations.

Discussion

To our knowledge, this study is the first to follow up a cohort in India, for a period of two years for COVID-19 infection and re-infection. In terms of surveillance of SARS-CoV-2 infection, though the nasopharyngeal swab has been the gold standard for diagnosis, the use of saliva

For peer review only

For peer review only - http://bmjopen.bmj.com/site/about/guidelines.xhtml

samples will be an acceptable alternate by the study participants as it is self-directed, non-invasive and has a good concordance with the nasopharyngeal swab. The study aims to address several gaps in the current scientific evidence of SARS-CoV-2 infection and immunity. Firstly, there are a limited number of studies that investigate the long term follow up of individuals for the rates of infection and re-infection in the community. Secondly, the study aims to look at the kinetics of IgG antibodies following infection. The cross-reactivity between SARS-CoV-2 and other human coronaviruses will support better understanding of determinants of symptomatic infection. The T cell and B cell memory responses would help in understanding the kinetics and longevity of immune responses in seropositive and seronegative individuals and would help in decision making with regard to booster vaccination. By studying the immunity and the risk of reinfection we can potentially understand the factors that contribute to symptomaticCOVID-19 infections. The study design also will allow the study of how the various VOC contribute to re-infections. Large scale vaccination had begun by the time enrolment had been completed. We anticipate that the majority of participants will be vaccinated at the end of the study and would have a hybrid immunity resulting from past infection and vaccine. In view of the one-year intensive follow up that requires weekly samples, we have planned to use salivary RT PCR and only symptomatic individuals will receive nasopharyngeal swab for RT PCR.

To conclude, CORES will help in estimating the re-infection rates, detailed immunogenicity amongst the COVID-19 positive individuals, establish the antibody kinetics and characterise the breakthrough infections amongst the vaccinated individuals in the community.

Contributors: The study design and concept were conceived by JJ and GK. RM will conduct the study as part of her PhD under the supervision of GK, SB, SP and JJ. JSP and JJ designed

For peer review only

421 the process evaluation and wrote the statistical analysis plan and JJ, DK and JSP organise data
422 management and will oversee field operations. PKH, RA, GSR performed the RT-PCR of the
423 weekly saliva samples. All authors provided edits and critiqued the manuscript for the scientific
424 content. All authors read and approved the final version of the manuscript.

425 **Competing interests:** The authors declare that they have no competing interests.

426 **Funding:** This study is funded by Bill and Melinda Gates Foundation funding INV-024915.
427 The funders have no role in the design of this study and will not have any role during its
428 execution, analyses, interpretation of the data, or decision to submit results.

429 **Consent for publication:** Not applicable.

430 **Acknowledgments:** We acknowledge the participants who are willing to participate in the
431 current study and help us in understanding the current knowledge gaps in COVID-19
432 infection and re-infections. We also thank the National Centre for Biological Sciences,
433 Bangalore for helping us in processing our weekly saliva samples.

434 **Abbreviations**

CAP	Chinnallapuram
CMC	Christian Medical College, Vellore
CMV	Cytomegalovirus
COVID-19	Coronavirus Disease-2019
CRF	Case Report Format
FRA	Field Research Assistant
hCoV	Human Coronavirus
LMIC	Low and Middle Income Country
NCBS	National Centre for Biological Sciences

For peer review only - http://bmjopen.bmj.com/site/about/guidelines.xhtml

PBMC	Peripheral Blood Mononuclear Cells
PPS	Probability proportionate to size
RT-PCR	Reverse Transcription -Polymerase Chain Reaction
SARS-COV-2	Severe acute respiratory syndrome coronavirus 2
SES	Socio Economic Status
ß-CoV	Betacoronavirus
VHDSS	Vellore Health & Demographic Surveillance System

References

1. Poland GA, Ovsyannikova IG, Kennedy RB. SARS-CoV-2 immunity: review and applications to phase 3 vaccine candidates. Lancet 2020;396:1595–606.

2. Hall V, Foulkes S, Insalata F, Kirwan P, Saei A, Atti A, et al. Protection against SARS-CoV-2 after Covid-19 Vaccination and Previous Infection. New England Journal of Medicine 2022;386:1207–20.

3. Dan JM, Mateus J, Kato Y, Hastie KM, Yu ED, Faliti CE, et al. Immunological memory to SARS-CoV-2 assessed for up to 8 months after infection. Science 2021;eabf4063.

4. Qureshi AI, Baskett WI, Huang W, Lobanova I, Hasan Naqvi S, Shyu CR. Reinfection With Severe Acute Respiratory Syndrome Coronavirus 2 (SARS-CoV-2) in Patients Undergoing Serial Laboratory Testing. Clinical Infectious Diseases 2022;74:294–300.

5. Chen X, Pan Z, Yue S, Yu F, Zhang J, Yang Y, et al. Disease severity dictates SARS-CoV-2-specific neutralizing antibody responses in COVID-19. Sig Transduct Target Ther 2020;5:1–6.

6. Long period dynamics of viral load and antibodies for SARS-CoV-2 infection: an observational cohort study | medRxiv [Internet]. [cited 2022 Apr 5];Available from: https://www.medrxiv.org/content/10.1101/2020.04.22.20071258v1.article-metrics

7. Rydyznski Moderbacher C, Ramirez SI, Dan JM, Grifoni A, Hastie KM, Weiskopf D, et al. Antigen-Specific Adaptive Immunity to SARS-CoV-2 in Acute COVID-19 and Associations with Age and Disease Severity. Cell 2020;183:996-1012.e19.

8. To KKW, Hung IFN, Ip JD, Chu AWH, Chan WM, Tam AR, et al. COVID-19 re-infection by a phylogenetically distinct SARS-coronavirus-2 strain confirmed by whole genome sequencing. Clin Infect Dis 2020;ciaa1275.

9. Tillett RL, Sevinsky JR, Hartley PD, Kerwin H, Crawford N, Gorzalski A, et al. Genomic evidence for reinfection with SARS-CoV-2: a case study. The Lancet Infectious Diseases 2021;21:52–8.

For peer review only

10. Bylicki O, Delarbre D, Mayet A, Ferrier A, Perisse A, Malle C, et al. Neutralizing antibody response to SARS-CoV-2 persists 9 months post symptom onset in mild and asymptomatic patients. Int J Infect Dis 2021;112:8–12.

11. Zhu L, Xu X, Zhu B, Guo X, Xu K, Song C, et al. Kinetics of SARS-CoV-2 Specific and Neutralizing Antibodies over Seven Months after Symptom Onset in COVID-19 Patients. Microbiol Spectr 2021;9:e0059021.

12. Cao WC, Liu W, Zhang PH, Zhang F, Richardus JH. Disappearance of antibodies to SARS-associated coronavirus after recovery. N Engl J Med 2007;357:1162–3.

13. Ng OW, Chia A, Tan AT, Jadi RS, Leong HN, Bertoletti A, et al. Memory T cell responses targeting the SARS coronavirus persist up to 11 years post-infection. Vaccine 2016;34:2008–14.

14. Gaunt ER, Hardie A, Claas ECJ, Simmonds P, Templeton KE. Epidemiology and Clinical Presentations of the Four Human Coronaviruses 229E, HKU1, NL63, and OC43 Detected over 3 Years Using a Novel Multiplex Real-Time PCR Method. J Clin Microbiol 2010;48:2940–7.

15. Callow KA, Parry HF, Sergeant M, Tyrrell DA. The time course of the immune response to experimental coronavirus infection of man. Epidemiol Infect 1990;105:435–46.

16. Braun J, Loyal L, Frentsch M, Wendisch D, Georg P, Kurth F, et al. SARS-CoV-2-reactive T cells in healthy donors and patients with COVID-19. Nature 2020;587:270–4.

17. Mateus J, Grifoni A, Tarke A, Sidney J, Ramirez SI, Dan JM, et al. Selective and cross-reactive SARS-CoV-2 T cell epitopes in unexposed humans. Science 2020;370:89–94.

18. Shaw RH, Stuart A, Greenland M, Liu X, Van-Tam JSN, Snape MD. Heterologous prime-boost COVID-19 vaccination: initial reactogenicity data. The Lancet 2021;397:2043–6.

19. Sun Q, Li J, Ren H, Pastor L, Loginova Y, Madej R, et al. Saliva as a testing specimen with or without pooling for SARS-CoV-2 detection by multiplex RT-PCR test. PLOS ONE 2021;16:e0243183.

Figure title

Figure 1: CORES study flowchart

For peer review only - http://bmjopen.bmj.com/site/about/guidelines.xhtml

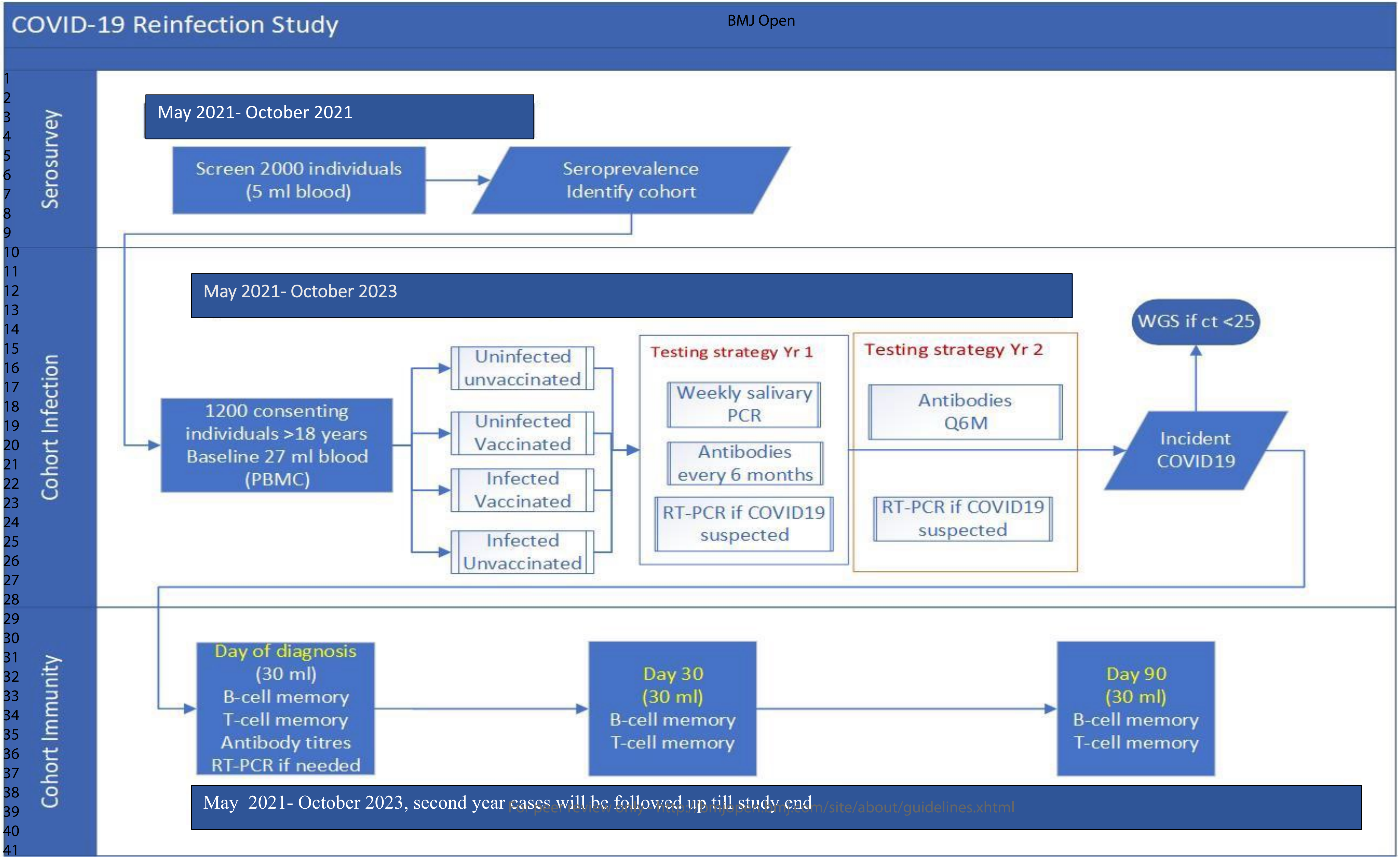
COVID-19 Reinfection Study
Serosurvey
May 2021- October 2021
Screen 2000 individuals (5 ml blood)
Seroprevalence Identify cohort
Cohort Infection
May 2021- October 2023
1200 consenting individuals >18 years Baseline 27 ml blood (PBMC)
Uninfected unvaccinated
Uninfected Vaccinated
Infected Vaccinated
Infected Unvaccinated
Testing strategy Yr 1
Weekly salivary PCR
Antibodies every 6 months
RT-PCR if COVID19 suspected
Testing strategy Yr 2
Antibodies Q6M
RT-PCR if COVID19 suspected
WGS if ct <25
Incident COVID19
Cohort Immunity
Day of diagnosis (30 ml) B-cell memory T-cell memory Antibody titres RT-PCR if needed
Day 30 (30 ml) B-cell memory T-cell memory
Day 90 (30 ml) B-cell memory T-cell memory
May 2021- October 2023, second year cases will be followed up till study end
